# Identification of the Students Learning Process During Education Robotics Activities

**DOI:** 10.3389/frobt.2020.00021

**Published:** 2020-03-13

**Authors:** David Scaradozzi, Lorenzo Cesaretti, Laura Screpanti, Eleni Mangina

**Affiliations:** ^1^Dipartimento di Ingegneria dell'Informazione (DII), Università Politecnica delle Marche, Ancona, Italy; ^2^LSIS - umr CNRS 6168, Laboratoire des Sciences de l'Information et des Systèmes, Equipe I&M (ESIL), Marseille, France; ^3^TALENT srl, Osimo, Italy; ^4^School of Computer Science, University College Dublin, Dublin, Ireland

**Keywords:** educational robotics, educational data mining, learning analytics, STEM activities assessment, learning process identification

## Abstract

This paper presents the design of an assessment process and its outcomes to investigate the impact of Educational Robotics activities on students' learning. Through data analytics techniques, the authors will explore the activities' output from a pedagogical and quantitative point of view. Sensors are utilized in the context of an Educational Robotics activity to obtain a more effective robot–environment interaction. Pupils work on specific exercises to make their robot smarter and to carry out more complex and inspirational projects: the integration of sensors on a robotic prototype is crucial, and learners have to comprehend how to use them. In the presented study, the potential of Educational Data Mining is used to investigate how a group of primary and secondary school students, using visual programming (Lego Mindstorms EV3 Education software), design programming sequences while they are solving an exercise related to an ultrasonic sensor mounted on their robotic artifact. For this purpose, a tracking system has been designed so that every programming attempt performed by students' teams is registered on a log file and stored in an SD card installed in the Lego Mindstorms EV3 brick. These log files are then analyzed using machine learning techniques (k-means clustering) in order to extract different patterns in the creation of the sequences and extract various problem-solving pathways performed by students. The difference between problem-solving pathways with respect to an indicator of early achievement is studied.

## Introduction

Educational Robotics (ER) has been widely used to support integrative STEM education because of its power to realize engaging multidisciplinary activities about science, technology, engineering, and mathematics, but also arts, language, and humanities (Mubin et al., 2013; Scaradozzi et al., 2019a,b). Furthermore, ER can also support inclusive education (Daniela and Lytras, 2019) and computer science and robotics literacy at all ages (Burbaite et al., 2013; Štuikys et al., 2013; Berry et al., 2016; Damaševicius et al., 2017; Vega and Cañas, 2019). Even if many studies explored ER to motivate students to learn, not all of them reported an evaluation of activities; those who focused on the evaluation of ER activities adopted qualitative (Denis and Hubert, 2001; Liu, 2010; Elkin et al., 2014), quantitative (Atmatzidou and Demetriadis, 2016; Kandlhofer and Steinbauer, 2016; Cesaretti et al., 2017; Scaradozzi et al., 2019c), or mixed methods approaches (Kim et al., 2015; Chalmers, 2018). In fact, in an ER activity (a lesson characterized by one or more ER exercises), students design, build, program, debug, and share their robotic artifacts; ER is based on the constructionist approach proposed by Papert (1980): when pupils create personal and meaningful products, they “build” knowledge in their mind. This kind of educational activity is characterized by a workflow, modeled by Martinez and Stager (2013) with the “Think Make Improve” (TMI) cycle, where three different phases are repeated cyclically:

- At the beginning, the educator proposes a problem to solve so students usually start thinking and designing their solution (“Think” phase);
- Then learners build their product: in an ER activity, it could be a hardware (a prototype) or software (a sequence of instructions) creation (“Make” phase);
- At the end of the construction phase, students start the robot, observe and analyze its behavior, debugging errors or trying to optimize the performance of the artifact (Improve phase): pupils have to examine carefully the feedback of the robot in order to decide the next designing or programming steps (so the cycle starts again with the Think phase).

The evaluation of a product created during a constructionist activity can be a challenging and time-consuming activity (Berland et al., 2014). Moreover, it is often based on the final product and not on the process underlying the designed task (Blikstein, 2011). However, new data mining and machine learning technologies allow researchers to capture detailed data related to problem-solving and programming trajectory of a large number of learners (Blikstein et al., 2014).

Recent studies (Berland et al., 2013; Blikstein et al., 2014; Chao, 2016; Wang et al., 2017; Bey et al., 2019; Filvà et al., 2019) have mostly applied machine learning techniques to data gathered from students during programming activities without the presence of physical robots, obtaining good results in the identification of different patterns in specific coding tasks (Table 1 summarizes machine learning techniques and features selected in these studies). Berland et al. (2013) and Chao (2016) used a k-means algorithm to discover patterns in the programming activity of novice programmers; the first study identified three general patterns (Tinkering, Exploring, and Refining) and presented a positive correlation between the quality of the programming sequences designed by the students and two of the emerged patterns (Tinkering and Refine). The second study represented the students' programming activity using five indicators and identified four clusters (sequent approach, selective approach, repetitious approach, and trial approach); the study showed that the performance was lower for learners in the trial approach cluster compared to the sequent and repetitious approach clusters. Blikstein et al. (2014) proposed two experiments using different machine learning techniques, trying to discover patterns in data collected from 370 undergraduate students and to predict their midterm and final exam grades. They obtained best results modeling students' programming trajectories using hidden Markov models and demonstrated that the group in which a student was clustered into was predictive of his or her midterm grade. Wang et al. (2017) used log data from Code.org^1^ and applied a long short-term memory recurrent neural network to predict students' future performance, obtaining good results in terms of accuracy and recall. Bey et al. (2019) identified three clusters in a dataset created collecting programs from 100 students registered on a 3-week course on the essential of Shell programming; they applied unsupervised clustering techniques (Hopkins statistic methods) for automatically identifying learners' programming behavior. Filvà et al. (2019) used the k-means technique on data generated by students' clicks in Scratch (and not on handpicked features), with the objective of categorizing learners' behavior in programming activities: they identified three different patterns and a strong correlation between these behaviors and the evaluation given by some teachers involved in the research project, using a rubric for programming assessment.

**Table 1:** Features and machine learning techniques of recent studies carried out in constructionist environments.

**Paper**	**Features selected in the experimentation**	**Machine learning techniques**
Blikstein et al. (2014)	[1st experiment] Code update differential, characterized by: number of lines added, lines deleted, lines modified, characters added, characters removed, characters modified.	[1st experiment] Simple regression between exam grades and average size of the code updates per student.
	[2nd experiment] Code update differential	[2nd experiment] X-means clustering algorithm.
	[3rd experiment] Code update differential	[3rd experiment] X-means clustering algorithm.
	[4th experiment] Code update curves (combination of frequency and size in changes made by students).	[4th experiment] Dynamic time warping and scaled dynamic time warping distance (to calculate the difference between two given code update curves).
	[5th experiment] Modeling of a student's trajectories as a hidden Markov model (HMM).	[5th experiment] k-medioid and hierarchical agglomerative clustering (to compute the different states of the HMM). Expectation maximization algorithm to compute both the transition and emission probabilities in the state diagram.
Berland et al. (2013)	Measures of individual program states (measures calculated for each program state) considering five features: action, logic, unique primitives, length, coverage.	X-Means clustering algorithm
Jormanainen and Sutinen (2012)	Six events: add statement, add command to code, remove line, upload program to robot, compiling errors, sum of all these events.	Decision trees, decision tables, Bayesian networks, and multilayer perceptrons to predict the students' progress. To measure the accuracy of the tested algorithms, they used the 10-fold cross-validation method.
Chao (2016)	Related to computational practice (five measures): sequence, selection, simple iteration, nested iteration, testing.	Ward's minimum variance method (to identify number of clusters), followed by the k-mean cluster analysis (on the identified cluster number).
Wang et al. (2017)	[1st experiment] A student's trajectory consists of all the program submissions, which are represented as ASTs (that contain all the information about a program and can be mapped back into a program). These ASTs are converted into program embeddings using a recursive neural network.	[1st experiment] Long Short-Term Memory (LSTM) Recurrent Neural Network (RNN).
	[2nd experiment] Same features of their 1st experiment, from ASTs they calculated program embeddings.	[2nd experiment] LSTM RNN
Bey et al. (2019)	Number of submissions, average time between two submissions, average number of changes, percentage of syntactical errors, time standard deviation (the standard deviation of the average time between two submissions), code standard deviation (the standard deviation of the average number of changes).	Mixture Gaussian Clustering algorithm
Filvà et al. (2019)	Clickstream	K-means cluster analysis

However, only one research study (to the best of our knowledge) applied machine learning to data collected during ER activities (Jormanainen and Sutinen, 2012); they did not collect data related to the programming sequences designed by the students but related to the pupils' interactions with the essential elements of the visual programming environment. Their system, using trees algorithm (J48 implementation), classified the students' activities into four classes, differentiating the observed students' group's progress with the purpose of identifying pupils with difficulties during the robot programming task. Ahmed et al. (2018) presented an interesting system that gives feedback to pupils in real time while they are programming the Lego Mindstorms EV3 robot^2^; they implemented a system (ROBIN) so that the Lego Mindstorms EV3 robot provided reflective feedback to pupils, transforming it into a learning companion: using ROBIN, students obtained advices based on the sequences created on the programming environments and based on the exercise proposed by the educator. But in this research project, the researchers did not train their system using machine learning techniques but using deterministic rules. The promising results obtained using machine learning techniques on data gathered from students during programming activities, and the lack of this type of study in the field of ER (Scaradozzi et al., 2019b), have prompted the research described in this paper. Thanks to an upgrade of the Lego Mindstorms EV3 programming blocks (implemented by the authors), it was possible to register some log files containing the programming sequences created by 353 Italian primary and secondary school students (organized in 85 teams) during the resolution of a robotics exercise related to the ultrasonic sensor. Integrating sensors allowed learners to obtain an interaction between the robot and the environment, but to effectively use these devices, they had to understand some key concepts about robotics and computer science, such as how to acquire and store data, how to cyclically repeat an acquisition (using loops), and how to create algorithms to obtain different robot's behaviors depending on the values detected by sensors (using conditional statements). The authors inputted the collected log files into k-means algorithms, with the purpose of verifying if there are different problem-solving patterns emerging from this dataset and of examining the interrelationships between the different problem-solving patterns and a performance indicator showing the students' team capability to reach a working program solution.

## Methods

### Procedure

**Figure 1:**
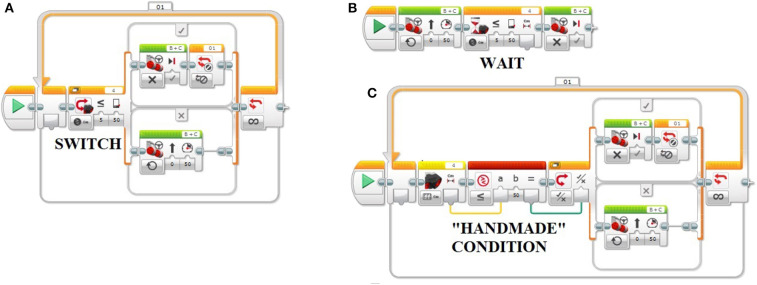
Three possible solutions for the exercise designed using the Lego Mindstorms EV3 software blocks: “Switch case” **(A)**, “Wait” **(B)**, and “Handmade condition” **(C)**.

At the first stage of this research project, authors implemented a *software modification* to the Lego Mindstorms EV3 Education Software blocks; thanks to this software development, every time that students tested their program on the robot Lego Mindstorms EV3, a “track” of the coding sequence was written in a log file stored in the SD card mounted on the robot. Fourteen schools participated in the experimentation, and the same protocol was performed for each of them. Firstly, an educator of TALENT srl (an Italian innovative startup involved in the research project) installed on the computers of the school the official Lego Mindstorms EV3 Education software and the update designed by the authors. An “Introduction to Robotics” course was then realized, taught in collaboration with TALENT; Constructionism (Papert, 1980) and problem-based learning (Savery, 2006) were the pedagogical approaches underlying the proposed course: during each lesson, students designed and created programming solutions to problems related to the robot. After a first part dedicated to the robot's actuators, the ultrasonic sensor was explored. An exercise was proposed by the instructor to the students: learners had to program the robot so that it stopped at a given distance from the wall, trying to be as precise as possible; they also had to consider a constraint: the maximum available time to design and test their coding solution (20 min for higher secondary school classes; 30 min for lower secondary and primary school classes). There are some elements that make this exercise quite tricky for novice students in robotics: they have to think about how to set the condition related to the ultrasonic sensor, how to use the iteration (loop block), and how to compensate the braking distance (the robot does not stop immediately when the EV3 brick sends the command “turn off” to the motors). Figure 1 shows three possible solutions to this problem: the simplest sequence (B) in terms of the number of blocks contains the Wait block; it makes the program wait for a condition becoming “true” before continuing to the next block in the sequence. The intermediate solution (A) contains the Switch block; this block is a container that can comprehend two or more sequences of programming blocks; a test at the beginning of the Switch determines which Case will run, and in this case, the test is designed on the ultrasonic sensor. The most complex solution (C) contains a “handmade” condition, created using a Sensor block (the yellow one) and a Compare block (the red one). Students' teams involved in the experimentation were free to design and test their programming solution (usually close to one of the sequences presented in Figure 1): the educator only explained the general meaning and the parameters of the useful blocks, and then the pupils started to work on their program.

At the end of the exercise, all the log files generated by the tracking system were downloaded from the SD card by the TALENT's educator and stored in the cloud storage.

The authors fed the collected log files (transformed into vectors thanks to a parsing system developed in Python) into a k-means algorithm, whose results provided clusters that represent different types of sequences designed by the students to solve the exercise. Then, for each team of the students involved in the experimentation, the number of sequences belonging to each cluster was calculated in order to get new features that characterized the students' programming activity (all the programming actions carried out by the participants with the intention to obtain the desired robot's behavior). These new features were used again as input data for a k-means algorithm, and different problem-solving behaviors emerged from this last step. An expert robotics educator defined for each log file the first working sequence created by the students' team, which allowed the educator to observe in which stage of the problem-solving process learners created their first working sequence. A working sequence is a program that can solve the exercise previously presented, and the conditions to be met are: correct conditional statement on the ultrasonic sensor and motors turned on using the right modality. Then, applying the formula:

$$\begin{array}{l} Indicator\;of\;early\;achievement=\frac{n{}^{\circ}\;of\;the\;first\;working\;sequence}{total\;tests\;number} \end{array}$$

Finally, a one-way non-parametric ANOVA (Kruskal–Wallis) test was conducted to examine the differences in the indicator according to the different problem-solving behaviors, which emerged from the machine learning technique. Moreover, the *post-hoc* Dunn test (Dunn, 1964), appropriate for groups with unequal numbers of observations (Zar, 2010), was employed to examine the significance of all possible pairwise comparisons among clusters.

### Participants

From March 2018 to September 2019, a total of 353 students from 14 Italian primary and lower/higher secondary schools (located in the Emilia Romagna and Marche regions) were involved in this study. Sixty-two students divided into 19 teams [Average Age (AA) = 17.29, Standard Deviation (SD) = 0.55] from school 1 were involved. School 2 had 22 students involved, divided into six teams (AA = 11.45, SD = 0.50). School 3 had 24 students involved, divided into six teams, but valid data were collected only from two of them (AA = 10.08, SD = 0.65). School 4 had 21 students involved, divided into five teams (AA = 11.70, SD = 0.47). School 5 had 19 students involved, divided into seven teams (AA = 11.63, SD = 0.83). School 6 had 25 students involved, divided into five teams (AA = 15.92, SD =0.28). School 7 had 24 students involved, divided into six teams, but valid data were collected only from three of them (AA = 12.00, SD = 0.46). School 8 had 23 students involved, divided into five teams (AA = 12.43, SD = 0.94). School 9 had 30 students, divided into six teams (AA = 9.63, SD = 0.53). School 10 had 26 students involved, divided into six teams (AA = 12.54, SD =0.51). School 11 had 19 students involved, divided into five teams (AA = 10.21, SD = 0.98). School 12 had nine students involved, divided into three teams (they were from lower secondary school, but no personal data were available). School 13 had 23 students involved, divided into six teams (AA = 11.87, SD = 1.29). School 14 had 26 students involved, divided into eight teams (AA = 10.24, SD = 0.83).

### Data Preparation

Students' teams designed 3,292 programming sequences to solve the robotics exercise previously described. Some technical steps are performed to transform these sequences into matrices; after this transformation, the following 12 indicators are calculated for each programming sequence. A function designed in Python realizes the parsing of the log file to calculate these 12 values.

- **Motors**: how many Motor blocks are contained in the sequence;
- **Loops**: how many Loop blocks are contained in the sequence;
- **Conditionals**: how many Conditional and Sensors blocks are contained in the sequence;
- **Others**: how many blocks are contained in the sequence belonging to different categories than Motors, Loops, and Conditionals;
- **Added**: how many blocks have been added, compared to the previous sequence;
- **Deleted**: how many blocks have been deleted, compared to the previous sequence;
- **Changed**: how many blocks have been changed, compared to the previous sequence;
- **Equal**: how many blocks have remained unchanged, compared to the previous sequence;
- **Delta Motors**: the amount of change in Motor blocks parameters, compared to the previous sequence (calculated only for blocks of the “Changed” category);
- **Delta Loops**: the amount of change in Loop blocks parameters, compared to the previous sequence;
- **Delta Conditionals**: the amount of change in Conditional blocks parameters, compared to the previous sequence;
- **Delta Others**: the amount of change in Other blocks parameters, compared to the previous sequence.

The authors decided to calculate the first four indicators (Motors, Loops, Conditionals, Others) because they represent the features of a sequence designed using the Lego Mindstorms EV3 software; moreover, they are key concepts in ER and computational curricula (Grover and Pea, 2013; Scaradozzi et al., 2015, 2019b; Allsop, 2019). Furthermore, as previously stated, an ER activity is characterized by a cyclical procedure for improving the programming sequence: for this reason, it is essential to calculate the differences between two contiguous sequences, represented by the last eight parameters (Added, Deleted, Changed, Equal, Delta Motors, Delta Loops, Delta Conditionals, and Delta Others). Each programming sequence designed by the learners is thus represented using these 12 indicators, and it can be considered as a point in the problem-solving trajectory (Berland et al., 2013) carried out by the students' team.

## Results

Clusters resulting from the application of k-means algorithm on programming sequences designed by the students' teams are shown in Table 2. Fourteen clusters were identified applying the Elbow Method (Kodinariya and Makwana, 2013), and their relation to teams' behavior is briefly reported.

Cluster 1: the team tested the same programming sequence several times (characterized by four blocks, similar to solution B in Figure 1); 32.99% of the sequences are categorized in this cluster.
Cluster 2: the team changed the condition and the threshold value for the ultrasonic sensor throughout the programming attempts (programming sequence similar to solution B in Figure 1); 3.25% of the sequences are categorized in this cluster.
Cluster 3: the team heavily changed the condition and the threshold value for the ultrasonic sensor (i programming sequence similar to solution B in Figure 1); 2.13% of the sequences are categorized in this cluster.
Cluster 4: the team refined the threshold value for the ultrasonic sensor and some parameters in a Motors block at the same time (in a programming sequence similar to solution A in Figure 1); 6.71% of the sequences are categorized in this cluster.
Cluster 5: the team refined both some parameters in a Motors block and some parameters in Others blocks (programming sequence similar to solution A in Figure 1); 0.18% of the sequences are categorized in this cluster.
Cluster 6: the team modified some parameters in a Loops block (in a programming sequence similar to solution A in Figure 1); 0.03% of the sequences are categorized in this cluster.
Cluster 7: the team heavily modified some parameters in a Motors block and refined the threshold value for the ultrasonic sensor (in a programming sequence similar to solution A or B in Figure 1); 1.64% of the sequences are categorized in this cluster.
Cluster 8: the team tested the same programming sequence (characterized by 11–12 blocks, similar to solution A or C in Figure 1 with the addition of Others block); 4.19% of the sequences are categorized in this cluster.
Cluster 9: the team tested the same programming sequence (characterized by eight to nine blocks, similar to solution A or B in Figure 1); 24.14% of the sequences are categorized in this cluster.
Cluster 10: the team refined the threshold designed for the ultrasonic sensor (programming sequence like solution A in Figure 1) and added two blocks; 4.04% of the sequences are categorized in this cluster.
Cluster 11: the team refined both the threshold for the ultrasonic sensor and some parameters in a Motors block and deleted two blocks (in a programming sequence similar to solution A in Figure 1); 4.04% of the sequences are categorized in this cluster.
Cluster 12: the team refined both the threshold designed for the ultrasonic sensor and some parameters in a Motors block (programming sequence similar to solution B in Figure 1, but with only one Motors block); 6.35% of the sequences are categorized in this cluster.
Cluster 13: the team refined both the threshold for the ultrasonic sensor and some parameters in a Motors block (in a programming sequence similar to solution B in Figure 1, with two Motors blocks); 9.39% of the sequences are categorized in this cluster.
Cluster 14: the team refined both the threshold designed for the ultrasonic sensor and some parameters in a Motors block (in this case, the sequence is extremely complex, characterized by 11–12 blocks, similar to solution C in Figure 1); 0.91% of the sequences belong to this cluster.

**Table 2:** Mean values and standard deviation values of the 12 indicators (reported in the section Data Preparation) calculated for each cluster, as presented in the section Results (M(SD)).

	**Same**	**Modified**	**Added**	**Deleted**	**DeltaMotors**	**DeltaLoops**	**DeltaConditionals**	**DeltaOthers**	**Motors**	**Loops**	**Conditionals**	**Others**
Cluster 1	3.80 (1.23)	0.00 (0.00)	0.04 (0.19)	0.03 (0.18)	0.00 (0.00)	0.00 (0.00)	0.00 (0.00)	0.00 (0.00)	0.89 (0.31)	0.21 (0.62)	1.99 (0.33)	1.03 (0.16)
Cluster 2	4.11 (1.54)	1.09 (0.29)	0.50 (0.71)	0.20 (0.40)	0.80 (3.87)	0.00 (0.00)	41.77 (8.82)	0.00 (0.00)	1.37 (0.61)	1.05 (1.18)	2.17 (0.40)	1.06 (0.23)
Cluster 3	4.39 (1.68)	1.14 (0.39)	0.63 (0.85)	0.30 (0.67)	0.93 (6.21)	0.00 (0.00)	101.92 (41.18)	0.00 (0.00)	1.49 (0.53)	1.20 (1.29)	2.36 (0.48)	1.11 (0.32)
Cluster 4	7.13 (1.03)	1.97 (0.36)	0.06 (0.24)	0.04 (0.19)	1.37 (5.00)	0.00 (0.00)	4.48 (6.26)	0.00 (0.00)	1.98 (0.13)	2.83 (0.60)	3.00 (0.15)	1.33 (0.60)
Cluster 5	7.50 (1.05)	2.83 (0.75)	0.00 (0.00)	0.00 (0.00)	6.67 (8.76)	0.00 (0.00)	0.60 (0.67)	20.00 (3.85)	2.00 (0.00)	3.00 (0.00)	3.00 (0.00)	2.33 (0.52)
Cluster 6	5.00 (0.00)	1.00 (0.00)	0.00 (0.00)	0.00 (0.00)	0.00 (0.00)	20.00 (0.00)	0.00 (0.00)	0.00 (0.00)	1.00 (0.00)	2.00 (0.00)	2.00 (0.00)	1.00 (0.00)
Cluster 7	3.78 (1.99)	1.44 (0.57)	0.31 (0.84)	0.11 (0.42)	58.78 (24.34)	0.00 (0.00)	14.51 (21.81)	0.02 (0.14)	1.37 (0.49)	0.76 (1.08)	2.13 (0.48)	1.06 (0.30)
Cluster 8	11.67 (1.91)	0.11 (0.38)	0.23 (0.58)	0.09 (0.32)	0.47 (2.33)	0.00 (0.00)	0.14 (0.91)	0.01 (0.12)	1.99 (0.12)	3.11 (0.79)	2.98 (0.19)	4.11 (1.07)
Cluster 9	8.51 (0.82)	0.03 (0.17)	0.04 (0.20)	0.04 (0.19)	0.06 (0.98)	0.00 (0.00)	0.03 (0.23)	0.00 (0.00)	1.92 (0.28)	2.67 (0.51)	2.64 (0.50)	1.16 (0.37)
Cluster 10	5.50 (1.80)	0.25 (0.50)	2.64 (1.13)	0.24 (0.51)	0.88 (4.72)	0.00 (0.00)	3.86 (11.23)	0.00 (0.00)	1.74 (0.56)	2.17 (0.96)	2.81 (1.02)	1.24 (0.66)
Cluster 11	5.96 (2.04)	0.37 (0.55)	0.32 (0.61)	2.30 (0.87)	1.67 (8.11)	0.00 (0.00)	6.10 (13.76)	0.00 (0.09)	1.08 (0.46)	1.99 (0.85)	2.05 (0.74)	1.33 (0.80)
Cluster 12	3.04 (0.66)	1.03 (0.18)	0.05 (0.21)	0.06 (0.23)	2.01 (6.03)	0.00 (0.00)	4.70 (6.47)	0.00 (0.00)	1.02 (0.14)	0.08 (0.40)	1.98 (0.25)	1.00 (0.00)
Cluster 13	4.64 (1.05)	0.17 (0.38)	0.05 (0.22)	0.03 (0.17)	0.05 (0.64)	0.00 (0.00)	0.56 (1.91)	0.00 (0.00)	2.02 (0.13)	0.13 (0.52)	2.03 (0.18)	1.00 (0.06)
Cluster 14	11.4 (1.79)	0.53 (0.90)	0.33 (0.84)	0.20 (0.55)	1.13 (6.21)	0.00 (0.00)	1.87 (7.34)	0.00 (0.00)	2.33 (0.48)	2.2 (0.66)	6.37 (0.61)	1.37 (0.49)

**Figure 2:**
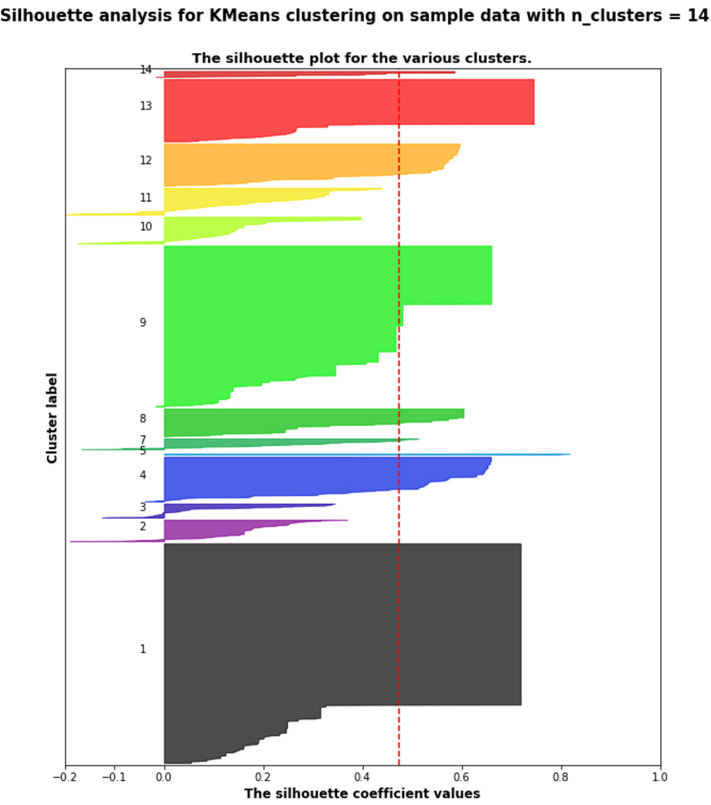
Silhouette scores for the 14 clusters (presented in the section Results) identified by the k-means algorithm.

Figure 2 presents the silhouette scores (Rousseeuw, 1987) for the 14 clusters identified by the k-means algorithm. Table 3 shows the Pearson correlation between these clusters and the indicator of early achievement: only cluster 3 shows a statistically significant positive correlation (Pearson coefficient correlation = 0.411, *p* < 0.0001); so teams that heavily changed the condition and the threshold value for the ultrasonic sensor did not obtain a working sequence in the first part of their work.

**Table 3:** Pearson correlation coefficient between clusters (see Table 2) and the indicator of early achievement.

**Cluster**	**Correlation**	***P*-value**
Cluster 3	0.411	< 0.0001
Cluster 13	0.124	0.259
Cluster 2	0.122	0.266
Cluster 11	0.014	0.897
Cluster 1	−0.006	0.956
Cluster 12	−0.009	0.936
Cluster 6	−0.024	0.826
Cluster 5	−0.024	0.826
Cluster 14	−0.037	0.735
Cluster 8	−0.049	0.654
Cluster 9	−0.057	0.602
Cluster 10	−0.082	0.453
Cluster 4	−0.088	0.421
Cluster 7	−0.122	0.267
Trials	−0.128	0.243

As previously stated in the section *Procedure*, after having clustered the students' programming sequences, the percentage of sequences belonging to each cluster was calculated for each group. Thus, the problem-solving process for each team was represented using a vector with 14 elements, the percentage of coding sequences in cluster 1, the percentage of coding sequences in cluster 2, etc. A matrix (size: 85 × 14) created considering these 14 features calculated for the 85 teams was then used as inputs for a k-means algorithm, with the aim of grouping teams with similar behavior. Applying again the Elbow Method (Kodinariya and Makwana, 2013), 10 different problem-solving pathways emerged (Table 4):

- Pathway 1: Prevalence of sequences belonging to cluster 9 and cluster 4; these teams designed a complex sequence (type A or C in Figure 1), generally refining the parameters, with a very low percentage of large changes in the condition or in the threshold for the ultrasonic sensor and implementing a quite high number of trials (18 teams in this cluster, 21.18%).
- Pathway 2: 17% of sequences belonging to clusters 3 and 4; these teams applied high changes in the condition and in the threshold designed for the ultrasonic sensor (eight teams in this cluster, 9.41%).
- Pathway 3: Prevalence of sequences belonging to cluster 13; these teams designed a compact sequence (type B in Figure 1) generally refining the threshold designed for the ultrasonic sensor and some parameters in a Motors block (eight teams in this cluster, 9.41%).
- Pathway 4: Prevalence of sequences belonging to cluster 1; these teams designed a compact sequence (type B in Figure 1) sometimes (14%) refining the threshold designed for the ultrasonic sensor and some parameters in a Motors block, sometimes (8%) applying high changes to the condition or to the threshold related to the ultrasonic sensor or to the Motors' parameters; a very high number of trials characterized this cluster (22 teams in this cluster, 25.88%).
- Pathway 5: Prevalence of sequences belonging to clusters 8 and 4; these teams designed a very complex sequence (type A or C in Figure 1), using also Others blocks and generally refining the parameters of the programming blocks (three teams in this cluster, 3.53%).
- Pathway 6: Relevant percentage (32%) of sequences belonging to clusters 10 and 11; these teams repeatedly deleted and added blocks to their sequence (similar to type A or B in Figure 1); a low number of trials characterized this cluster (13 teams in this cluster, 15.29%).
- Pathway 7: Prevalence of sequences belonging to clusters 8 and 4; the team designed a complex sequence using also four Others blocks (type A in Figure 1), generally refining the parameters, without any sequence with large changes in the condition or in the threshold for the ultrasonic sensor; this team also experimented some simple sequences (cluster 1, type B in Figure 1) (one team in this cluster, 1.18%).
- Pathway 8: Prevalence of sequences belonging to cluster 14; these teams designed the most complex sequences (type C in Figure 1), generally refining the parameters, without any sequence with large changes in the condition or in the threshold for the ultrasonic sensor (two teams in this cluster, 2.35%).
- Pathway 9: Relevant percentage (32%) of sequences belonging to clusters 10 and 11; the team repeatedly deleted and added blocks to their sequence (similar to type A in Figure 1) and repeatedly changed parameters in the programming blocks (36% of sequences in cluster 4); a low number of trials characterized this cluster (one team in this cluster, 1.18%).
- Pathway 10: the lowest number of trials (18) and a relevant percentage (11%) of sequences in cluster 7 (high changes in Motors parameters) characterized these teams (nine teams in this cluster, 10.59%).

**Table 4:** mean (M) and standard deviation (SD) of the 10 pathways (P) resulting from the second clustering algorithm (M(SD)).

	**Cluster 1**	**Cluster 2**	**Cluster 3**	**Cluster 4**	**Cluster 5**	**Cluster 6**	**Cluster 7**	**Cluster 8**	**Cluster 9**	**Cluster 10**	**Cluster 11**	**Cluster 12**	**Cluster 13**	**Cluster 14**	**Trials**
P1	0.04 (0.05)	0.02 (0.03)	0.02 (0.02)	0.24 (0.17)	0.00 (0.00)	0.00 (0.00)	0.00 (0.00)	0.00 (0.01)	0.56 (0.20)	0.05 (0.03)	0.05 (0.04)	0.00 (0.01)	0.01 (0.02)	0.01 (0.03)	45.67 (25.53)
P2	0.25 (0.27)	0.07 (0.05)	0.1 (0.03)	0.04 (0.05)	0.00 (0.00)	0.00 (0.00)	0.00 (0.01)	0.00 (0.00)	0.27 (0.23)	0.05 (0.05)	0.05 (0.04)	0.01 (0.02)	0.16 (0.24)	0.00 (0.00)	33.38 (17.94)
P3	0.07 (0.07)	0.08 (0.11)	0.00 (0.00)	0.00 (0.00)	0.00 (0.00)	0.00 (0.00)	0.01 (0.04)	0.00 (0.00)	0.05 (0.12)	0.00 (0.01)	0.01 (0.03)	0.01 (0.02)	0.76 (0.23)	0.00 (0.00)	31.75 (19.75)
P4	0.72 (0.16)	0.04 (0.03)	0.02 (0.01)	0.01 (0.03)	0.00 (0.00)	0.00 (0.00)	0.02 (0.03)	0.00 (0.00)	0.02 (0.05)	0.01 (0.02)	0.01 (0.02)	0.14 (0.15)	0.02 (0.05)	0.00 (0.00)	56.95 (30.75)
P5	0.00 (0.00)	0.00 (0.00)	0.00 (0.00)	0.09 (0.15)	0.00 (0.00)	0.00 (0.00)	0.00 (0.00)	0.81 (0.16)	0.01 (0.02)	0.03 (0.03)	0.06 (0.02)	0.00 (0.00)	0.00 (0.00)	0.00 (0.00)	42.67 (36.14)
P6	0.17 (0.11)	0.02 (0.03)	0.01 (0.02)	0.02 (0.06)	0.00 (0.00)	0.00 (0.00)	0.01 (0.02)	0.00 (0.00)	0.42 (0.12)	0.18 (0.07)	0.14 (0.06)	0.01 (0.02)	0.03 (0.09))	0.00 (0.00)	22.15 (15.02)
P7	0.05 (0.00)	0.00 (0.00)	0.00 (0.00)	0.13 (0.00)	0.11 (0.00)	0.00 (0.00)	0.02 (0.00)	0.47 (0.00)	0.16 (0.00)	0.04 (0.00)	0.02 (0.00)	0.00 (0.00)	0.00 (0.00)	0.00 (0.00)	55.00 (0.00)
P8	0.05 (0.07)	0.00 (0.00)	0.00 (0.00)	0.00 (0.00)	0.00 (0.00)	0.00 (0.00)	0.00 (0.00)	0.00 (0.00)	0.03 (0.04)	0.11 (0.06)	0.09 (0.05)	0.02 (0.04)	0.03 (0.04)	0.67 (0.02)	18.00 (2.83)
P9	0.12 (0.00)	0.00 (0.00)	0.00 (0.00)	0.36 (0.00)	0.00 (0.00)	0.04 (0.00)	0.00 (0.00)	0.00 (0.00)	0.16 (0.00)	0.12 (0.00)	0.2 (0.00)	0.00 (0.00)	0.00 (0.00)	0.00 (0.00)	25.00 (0.00)
P10	0.20 (0.17)	0.01 (0.02)	0.00 (0.01)	0.02 (0.04)	0.00 (0.00)	0.00 (0.00)	0.11 (0.04)	0.00 (0.01)	0.27 (0.29)	0.08 (0.03)	0.05 (0.05)	0.04 (0.05)	0.21 (0.25)	0.00 (0.00)	18.22 (8.42)

*Columns report clusters obtained from the first clustering technique (as illustrated in the section Results). Trials represents the mean number of tests carried out by the team of students*.

**Figure 3:**
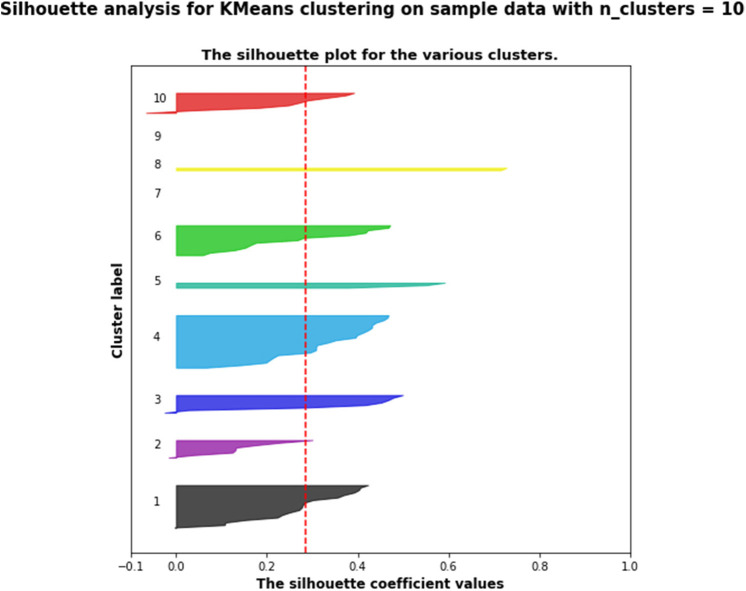
Silhouette scores for the 10 pathways (presented in the section Results) identified by the k-means algorithm.

**Figure 4:**
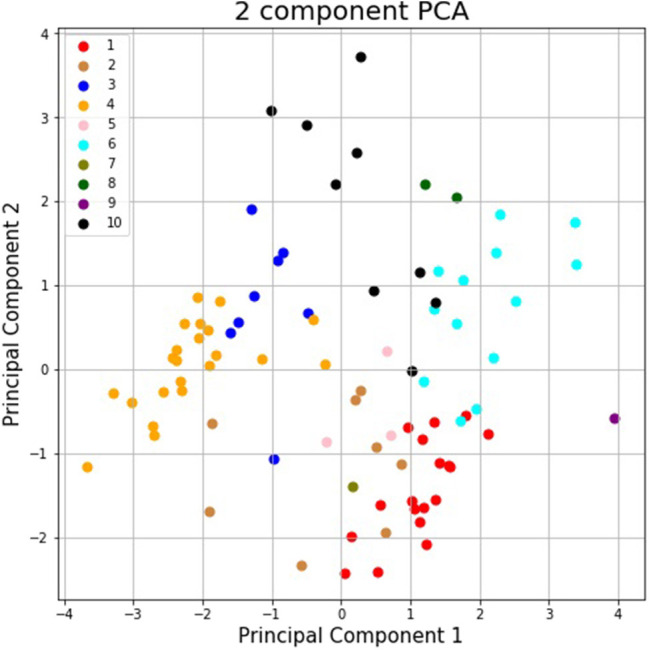
Representation of the identified pathways implemented by the students' teams along two principal components, calculated according to the PCA approach.

Figure 3 shows the silhouette scores for the 10 pathways presented above; Figure 4 is obtained after applying a two-dimensional principal component analysis (PCA): it presents the distribution of the identified pathways implemented by the students' teams along two principal components, calculated according to the PCA approach.

**Figure 5:**
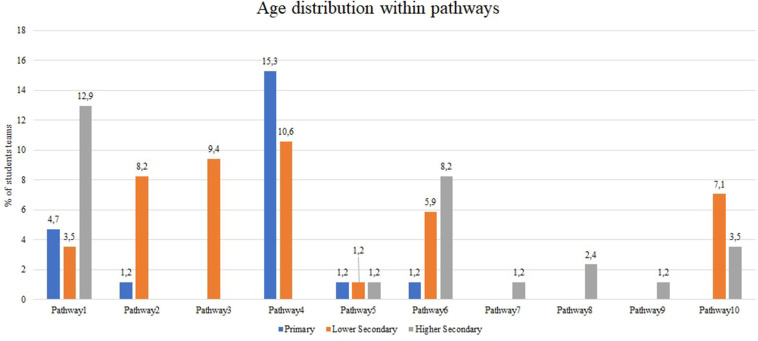
The age-related differences between the students' teams involved in the experimentation, within the 10 problem-solving pathways.

Figure 5 presents the age-related differences between the students' teams involved in the experimentation, within these 10 pathways: the majority of the higher school students adopted pathways 1 (a complex sequence with some refinements of the programming parameters) and 6 (a complex sequence with considerable variation to the condition set for the ultrasonic sensor); the majority of the lower school students adopted pathways 3 (a compact sequence with a refinement of the programming parameters) and 4 (a compact sequence with considerable variation to the condition set for the ultrasonic sensor); the majority of the primary school students adopted pathway 4 (a compact sequence with considerable variation to the condition set for the ultrasonic sensor).

**Figure 6:**
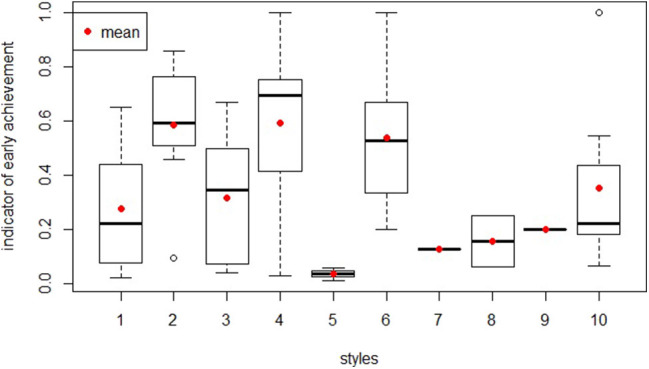
Distributions of the indicator of early achievement in the 10 selected pathways.

Figure 6 shows the distributions of the indicator of early achievement in the 10 selected cluster. Excluding those problem-solving behaviors that were shown by less than three groups (styles 7, 8, 9) from the analysis, significant differences (chi-square = 25.54, *p* = 0.0002711, d_f_ = 6) were found among the seven different clusters of group behavior. Pairwise comparisons using Dunn's test for multiple comparisons of independent samples with Bonferroni's *P*-value adjustment method showed that significant differences could be found between clusters 1 and 4 (*p* = 0.014), 2 and 5 (*p* = 0.035), and 4 and 5 (*p* = 0.016).

## Discussion

This brief research report presents an innovative application of machine learning techniques in the field of ER, for the identification of different problem-solving pathways and the analysis of how students learn to utilize sensors during an ER activity. The k-means algorithm identified 10 “pathways” that marked the students' teams' programming activity, during the resolution of specific exercise related to the ultrasonic sensor. Analyzing the pathways presented in the previous section, two main approaches to programming emerged: some teams modify the blocks' parameters implementing small changes, moving toward their objective by “small steps” (pathways 1, 3, 5); other teams design high changes (frequently modifying the symbol in the condition for the ultrasonic sensor, applying considerable variation to the threshold set for the ultrasonic sensor, repeatedly deleting/adding blocks, etc.) to their programming blocks from one test to another (pathways 2, 4, 6). The majority of the groups showing the first incremental approach (pathways 1, 3, 5) reached a working sequence during their first testing stage (an indicator of early achievement <0.4), unlike the teams with the “high changes approach” (pathways 2, 4, 6). This is a similar result compared to Blikstein et al. (2014), who identified that a “steadier incremental steps” strategy of programming correlated to a better performance in the resolution of the exercise. Pathway 4, with the highest number of trials (57) (Table 2), contains teams that did not obtain a working sequence in their first part of their work, and this result is similar to Chao (2016) but opposed to Filvà et al. (2019).

Future work of this study includes the analysis of more extensive set of challenges in order to obtain more general results. The dataset considered in this paper is quite small (in particular, for pathways 7, 8, and 9): ER is an approach characterized by teamwork, so despite having involved 353 primary and secondary school students in the experimentation, we obtained valid data from 85 teams (participants were divided into teams of three to four members who worked together to design software solutions). The promising results of this preliminary study have encouraged the authors to involve new classes in the experimentation in order to continue the validation of the approach. The authors intend also to utilize a recurrent neural network, in particular, the long short-term memory autoencoders (a structure specifically designed to support sequences of input data Hochreiter and Schmidhuber, 1997), in order to translate the programming sequences created by the students into fixed-length vectors (compress representation of the input data), maintaining a high level of information content. As a result, these vectors obtained from the autoencoders compression will be used as input features for supervised and/or unsupervised algorithms. Another possible approach that the authors intend to use for the same task (dimensionality reduction) is the PCA.

## Data Availability Statement

The datasets generated for this study are available on request to the corresponding author.

## Ethics Statement

Ethical review and approval were not required for the study on human participants in accordance with the local legislation and institutional requirements. Written informed consent was obtained from the participants' legal guardian/next of kin.

## Author Contributions

DS coordinated the research project and revised the paper. LC conceived the presented idea, designed the tracking system, implemented the machine learning analysis of the data, and wrote the paper, in collaboration with LS. LS developed the statistical analysis. EM supervised the machine learning analysis and revised the paper.

### Conflict of Interest

LC was employed by the company TALENT srl. The remaining authors declare that the research was conducted in the absence of any commercial or financial relationships that could be construed as a potential conflict of interest.

## Footnotes

^1^https://code.org

^2^https://education.lego.com/en-us/middle-school/intro/mindstorms-ev3
